# Estimated Divergence Times of *Lecanicillium* in the Family Cordycipitaceae Provide Insights Into the Attribution of *Lecanicillium*

**DOI:** 10.3389/fmicb.2022.859886

**Published:** 2022-05-06

**Authors:** Ye-Ming Zhou, Jun-Rui Zhi, Jiao-Jiao Qu, Xiao Zou

**Affiliations:** ^1^Institute of Entomology, Guizhou University, Guiyang, China; ^2^Institute of Fungus Resources, Guizhou University, Guiyang, China; ^3^College of Tea Sciences, Guizhou University, Guiyang, China

**Keywords:** entomopathogenic fungi, *Lecanicillium* spp., new species, origin and evolution, phylogeny

## Abstract

**Background:**

The genus *Lecanicillium* W.Gams & Zare is a recognized insect pathogen but members of the genus have been found parasitizing various hosts including arthropods, nematodes, plants, and fungi. The new classification system for fungi proposed to reject *Lecanicillium* and transfer some of the species to the genus *Akanthomyces*. However, the attribution problem of most species in the original genus *Lecanicillium* remains unsolved. The current study aimed to improve understanding of the pivotal internal phylogeny in *Lecanicillium* by estimating the divergence times of *Lecanicillium* to provide additional insights into the status of this genus within the family Cordycipitaceae.

**Results:**

Dating analyses support the supposition that the ancestor of *Lecanicillium* was in the Cretaceous period (84.36 Mya, 95% HPD: 72.12–94.74 Mya). After originating from a common ancestor, eight clades of *Lecanicillium* were derived and evolved independently in parallel with other genera of Cordycipitaceae. Based on the clear divergence age estimates, *Lecanicillium* clade 8 originated earlier as an independent group in the Cretaceous period (75.61 Mya, 95% HPD: 63.31–87.54 Mya), while *Lecanicillium* clades 1–7 originated later as an independent group in the boundary of the Cretaceous and Paleogene periods (64.66 Mya, 95% HPD: 52.75–76.74 Mya). *Lecanicillium huhutii* formed an independent branch in a polytomy together with a clade containing *Lecanicillium tenuipes* (BI posterior probabilities 1, ML bootstrap 100%).

**Conclusion:**

The pivotal internal phylogeny, origin, and evolutionary history of *Lecanicillium* in the family Cordycipitaceae were investigated. Phylogenetic and morphological analyses indicated that there are eight representative clades (four representative branches of evolutionary history), including clade 1 (members have a relatively uniform sporulation structure comprising globose heads with a higher number of conidia), clade 8 (including all members of *Gamszarea*), clades 2–5 (the differences of the divergence time estimations were smaller compared with other clades), and clade 6–7 (members are close to *Gibellula*, *Hevansia*, and *Ascopolyporus*). Based on the above findings, a novel spider-pathogenic fungus, *Lecanicillium huhutii*, is described. All other species in *Lecanicillium* clade 1 (*Lecanicillium araneogemum*, *L. nodulosum*, *L. pissodis*, and *L. uredinophilum*) should be transferred to the genus *Akanthomyces*. Furthermore, the monotypic genus *Parengyodontium* should be merged with the genus *Gamszarea*. More novel species need to be discovered to thoroughly resolve the attribution problem of *Lecanicillium*. Finally, no major lineages of *Lecanicillium* were correlated with the nearby Cretaceous-Tertiary extinction event, indicating that the diversity of *Lecanicillium* is more likely to be caused by long-term environmental adaptation and coevolution with insects rather than by dramatic extinction events.

## Introduction

Although members of the genus have been found parasitizing various hosts including arthropods, nematodes, plants, and fungi, the genus *Lecanicillium* W. Gams and Zare is recognized and largely known as an insect pathogen (Goettel et al., 2008; Sukarno et al., 2009; Su et al., 2019). Members of the genus *Lecanicillium* have the potential for development as effective biological control agents against numerous plant diseases, insect pests, and plant-parasitic nematodes (Goettel et al., 2008). To date, approximately 15 commercial preparations based on *Lecanicillium* spp. have been, or are currently being developed (Faria and Wraight, 2007). Species of the genus *Lecanicillium* are characterized by the formation of slender phialides, mostly from procumbent or prostrate aerial hyphae, singly or in terminal and intercalary whorls, and erect conidiophores with one or several whorls of phialides may also occur (Zare and Gams, 2001). This genus was established to accommodate entomogenous and fungicolous verticillium-like anamorphs of the family Clavicipitaceae (order Hypocreales) previously classified in *Verticillium* section *Prostrata* (Gams and Zare, 2001). The genus is typified by *Lecanicillium lecanii* with *Torrubiella confragosa* as the sexual morph. All insect pathogens formerly included in *Verticillium* were reclassified in this genus (Zare and Gams, 2001; Wijayawardene et al., 2017). Contemporaneously, the genus *Simplicillium* W. Gams and Zare was established to accommodate some species that were similar to those of the genus *Lecanicillium* but formed a distinct monophyletic group based on molecular data (Zare and Gams, 2001).

Sung et al. (2007) demonstrated the phylogenetic classification of *Cordyceps* and the Clavicipitaceous fungi and divided the Clavicipitaceae into Clavicipitaceae, Cordycipitaceae, and Ophiocordycipitaceae. Therefore, *Lecanicillium* was transferred into the family Cordycipitaceae along with other asexual genera like *Beauveria* Vuill., *Akanthomyces* Lebert., and *Simplicillium*. This work supported the findings of Zare and Gams (2001) that *Lecanicillium* is paraphyletic and morphological characters of these genera, especially the phialides and conidia, are difficult to distinguish a similar taxonomic group (Zare and Gams, 2008; Sukarno et al., 2009).

Kepler et al. (2017) revisited the taxonomic affinities of the family Cordycipitaceae (order Hypocreales) and resolved competing names based on the principles of priority, recognition of monophyletic groups, and the practical use of affected taxa. The type species of *Lecanicillium* was placed within the genus *Akanthomyces*, creating a conflict between the usage of the two generic names. In accordance with the reframing of Article 59 of the International Code of Nomenclature for algae, fungi, and plants (ICN) (McNeill et al., 2012). Kepler et al. (2017) proposed to maintain nine generic names, including *Akanthomyces* Lebert, *Ascopolyporus* Möller, *Beauveria* Vuill., *Cordyceps* Fr., *Engyodontium* de Hoog, *Gibellula* Cavara, *Hyperdermium* J.F. White, R.F. Sullivan, Bills and Hywel-Jones, *Parengyodontium* C.C. Tsang, J.F.W. Chan, W.M. Pong, J.H.K. Chen, A.H.Y. Ngan, Cheung, C.K.C. Lai, D.N.C. Tsang, S.K.P. Lau, and P. C.Y. Woo, and *Simplicillium* Gams and Zare, and two new genera including *Blackwellomyces* Spatafora and Luangsa-ard and *Hevansia* Luangsaard, Hywel-Jones and Spatafora (Kepler et al., 2017). Eight generic names were rejected, including *Evlachovaea* B.A. Borisov and Tarasov, *Granulomanus* de Hoog and Samson, *Isaria* Pers., *Lecanicillium* W. Gams and Zare, *Microhilum* H.Y. Yip and A.C. Rath, *Phytocordyceps* C.H. Su and H.H. Wang, *Synsterigmatocystis* Constantin, and *Torrubiella* Boud.

Currently, 35 species of the genus *Lecanicillium* have been formally described and are listed in the Index Fungorum.^1^ However, only five of these species were transferred to *Akanthomyces* by Kepler et al. (2017), and the attribution of most species in the original genus *Lecanicillium* remains an unsolved problem. Moreover, some novel species described after Kepler’s proposal were still placed in the genus *Lecanicillium*, such as *L. cauligalbarum* (Zhou et al., 2018), *L. coprophilum* (Su et al., 2019), *L. gracile* (Ponizovskaya et al., 2020), and *L. praecognitum* (Crous et al., 2020). Consequently, there is a question as to whether all members of the genus *Lecanicillium* should be transferred into the genus *Akanthomyces* or whether more new genera should be established, or whether there is an alternative solution to accommodate other species of *Lecanicillium*? Some scholars have made attempts to address this question. For example, Zhang et al. (2020) transferred five species of the genus *Lecanicillium* (*L. coprophilum*, *L. kalimantanense*, *L. restrictum*, *L. testudineum*, and *L. wallacei*) to a new genus, *Gamszarea* (for contributions of Walter Gams and Rasoul Zare to the taxonomic study of *Lecanicillium* W. Gams and Zare) based on ITS and multilocus (*TEF*, *RPB1*, *RPB2*, *LSU*, and *SSU*) sequence data. In addition, Wang et al. (2020) transferred three species of *Lecanicillium* (*L. acerosum*, *L. primulinum*, and *L. subprimulinum*) to a new genus, *Flavocillium*, based on ITS sequence data. Further work is needed to attempt to fully resolve the attribution problem of *Lecanicillium*.

New generic names for these species in the family Cordycipitaceae need introducing, supported by more detailed morphological and phylogenetic evidence combined with a larger sampling of taxa (Wang et al., 2020). Phylogenetic analysis revealed that *LSU* and *SSU* could not be used for identifying the strains alone because of the small interspecific differences (Zhou et al., 2020). The current study was performed to obtain a better understanding of the pivotal internal phylogeny in *Lecanicillium*, to execute accurate phylogenetic research, to estimate evolutionary timescales using the molecular clock, and to improve understanding of evolutionary processes across all taxonomic levels. A phylogenetic analysis was conducted with four loci (ITS, *TEF*, *RPB1*, and *RPB2*) from 97 species, including almost all species of the genus *Lecanicillium*. The calibration point in the molecular clock was based on the fossil record of *Paleoophiocordyceps coccophagus* G. H. Sung, Poinar, and Spatafora, a fungal animal parasite that had morphological features similar to the asexual states of *Hirsutella* and *Hymenostilbe* and belonged to the family Ophiocordycipitaceae (Sung et al., 2008). In addition, a detailed characterization of a novel species of the genus *Lecanicillium* was performed.

## Materials and Methods

### Specimen Collection and Fungus Isolation

The novel asexual morph species of *Lecanicillium* was isolated from spider cadavers hidden in a hole of a tree in Huaxi park of Guizhou province, China, on 5 October 2013. The isolated strains GZUIFRhuhu and GZUIFRhuhu1 were deposited at the Institute of Fungal Resources of Guizhou University (GZAC).

### Strain Culture and Observations

Isolated strains were inoculated on potato dextrose agar (PDA) at 25°C for 20 days under 12-h light/12-h dark conditions. For optical microscopy observations and imaging, fresh hyphae were stained with lactophenol cotton blue solution or normal saline, and the samples were observed with an optical microscope (BK5000, OPTEC, United States).

### DNA Extraction, PCR Amplification, and Sequencing

Genomic DNA was extracted using a previously described method (Chiriví-Salomón et al., 2015; Zou et al., 2016). PCR amplification of the ITS region, *TEF*, *RPB1*, and *RPB2* used primers and PCR conditions described in previous research (Zhou et al., 2018). For phylogenetic analysis of *Lecanicillium*, sequences of selected taxa based on recent phylogenetic studies of *Lecanicillium* (Crous et al., 2020; Ponizovskaya et al., 2020; Wang et al., 2020; Zhang et al., 2020), *Akanthomyces*, and other genera of Cordycipitaceae (Sung et al., 2007; Kepler et al., 2017; Mongkolsamrit et al., 2018) were downloaded from the National Center for Biotechnology Information (NCBI) databases.^2^ A total of 135 accessions were selected for this study (Supplementary Table 1).

### Sequence Alignment and Phylogenetic Analyses

The DNA sequences used in this study were edited using LASERGENE software (version 6.0; DNASTAR, Madison002C WI, United States). Multiple sequence alignments for *TEF*, *RPB1*, and *RPB2* were performed in MAFFT (Katoh et al., 2009) with the default settings. Multiple sequence alignments for ITS were conducted using the MUSCLE algorithm (Edgar, 2004) from MEGA 6 (Tamura et al., 2013). A multiple alignment of the combined partial ITS + *TEF* + *RPB1* + *RPB2* sequences was assembled with MEGA 6 (Tamura et al., 2013) and SEQUENCEMATRIX 1.7.8 (Vaidya et al., 2011). The command “hompart” in PAUP* 4.0b10 was used for the assessment of concordance amongst the genes and the ITS region (Swofford, 2001). Bayesian inference (BI) was performed using MRBAYES 3.2 (Ronquist et al., 2012) and maximum likelihood (ML) analysis was performed using RAxML (Muvea et al., 2014) to analyze the combined data, which were divided into twelve separate partitions (Kepler et al., 2017; Mongkolsamrit et al., 2018). Nucleotide substitution models were determined by MrModeltest 2.3 (Nylander et al., 2004). For BI, 10,000,000 generations were performed with one tree selected every 500th generation and the GTR + I + G evolutionary model was used. For ML, the model GTRGAMMA was used and a bootstrap analysis with 500 replicates was performed to assess statistical support for the tree topology. Phylogenetic trees were viewed with TREEGRAPH.

### Divergence Time Estimation of *Lecanicillium* in Cordycipitaceae

The Bayes MCMC algorithm was performed to estimate divergence time with the BEAST v. 2.4.5 software package (Bouckaert et al., 2014). An evolutionary event was used in the analysis as a soft constraint following a uniform limitation of one internal calibration point corresponding to the fossil record *P. coccophagus*, which was a fungal parasite of a scale insect from the Cretaceous period (99–105 Mya) (Sung et al., 2008; Berbee and Taylor, 2010). The tree topology was estimated by RAxML in the final step of the process. The data by gene was partitioned using the general time reversible (GTR) substitution model for each partition with jModelTest v. 2.1.7. A relaxed clock log normal model was used for BEAST. BEAST analyses were run for 50,000,000 generations, logging parameters, and trees every 1,000 generations. Convergence, mixing, and the effective sample sizes (ESS) of parameters were checked in Tracer v.1.6.5 (Zhang et al., 2018). Three repeat analyses were performed for accuracy and LogCombiner (Bouckaert et al., 2014) was used to combine the runs. The first 20% of trees representing the starting and unreliable results were removed from the analysis and a maximum clade tree was created with TreeAnnotator v. 2.4.5 (Bouckaert et al., 2014).

## Results

### Phylogenetic Analyses

**FIGURE 1:**
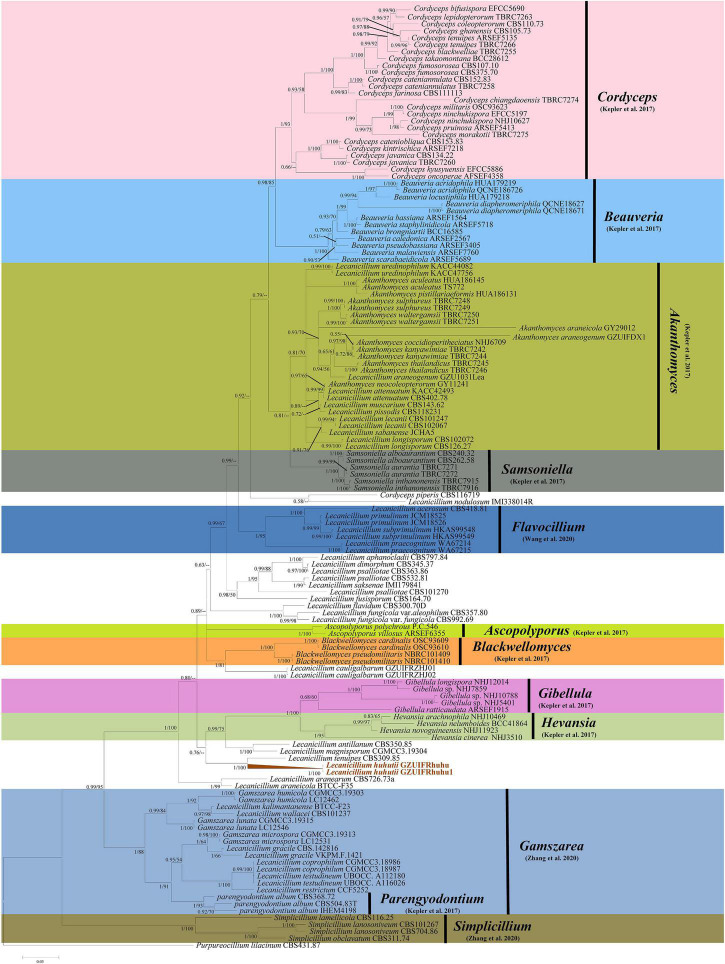
Phylogenetic relationships of the form genera *Lecanicillium*, *Cordyceps*, *Beauveria*, *Akanthomyces*, *Samsoniella*, *Flavocillium*, *Ascopolyporus*, *Blackwellomyces*, *Gibellula*, *Hevansia*, *Gamszarea*, *Parengyodontium*, and *Simplicillium* in the family Cordycipitaceae. Numbers near the nodes (bootstrap percentage values) correspond to the Bayesian posterior probabilities (BPP) and the ML methods. Bootstrap percentage values are based on 1,000,000 replicates and only the bootstrap proportions ≥ 0.50 are provided. ML values < 50% are shown as “–.” The tree is drawn to scale, with branch lengths in the same units as those of the evolutionary distances used to infer the phylogenetic tree.

The sequence data set consisted of 1783 characters, including inserted gaps (ITS: 511 bp; *TEF*: 404 bp; *RPB1*: 499 bp; *RPB2*: 369 bp). There was no noticeable difference in topology between the BI and ML phylogenies and their topologies reflected the same evolutionary relationships. The trees were reconstructed with almost all species of the genus *Lecanicillium* (only *Lecanicillium evansii* could not be found in the NCBI database). All major clades in Cordycipitaceae were strongly supported with ML bootstrap proportion and Bayesian posterior probabilities (Figure 1) and the overall topology of the best tree generated in ML analysis was essentially congruent with previous phylogenetic studies of Cordycipitaceae. *Lecanicillium huhutii* formed an independent branch in a polytomy together with a clade containing *L. tenuipes* (BI posterior probabilities 1, ML bootstrap 100%). The *L. huhutii* lineage received maximum statistical support (BI posterior probabilities 1, ML bootstrap 100%).

### Dating and Evolution of *Lecanicillium* in the Family Cordycipitaceae

**FIGURE 2:**
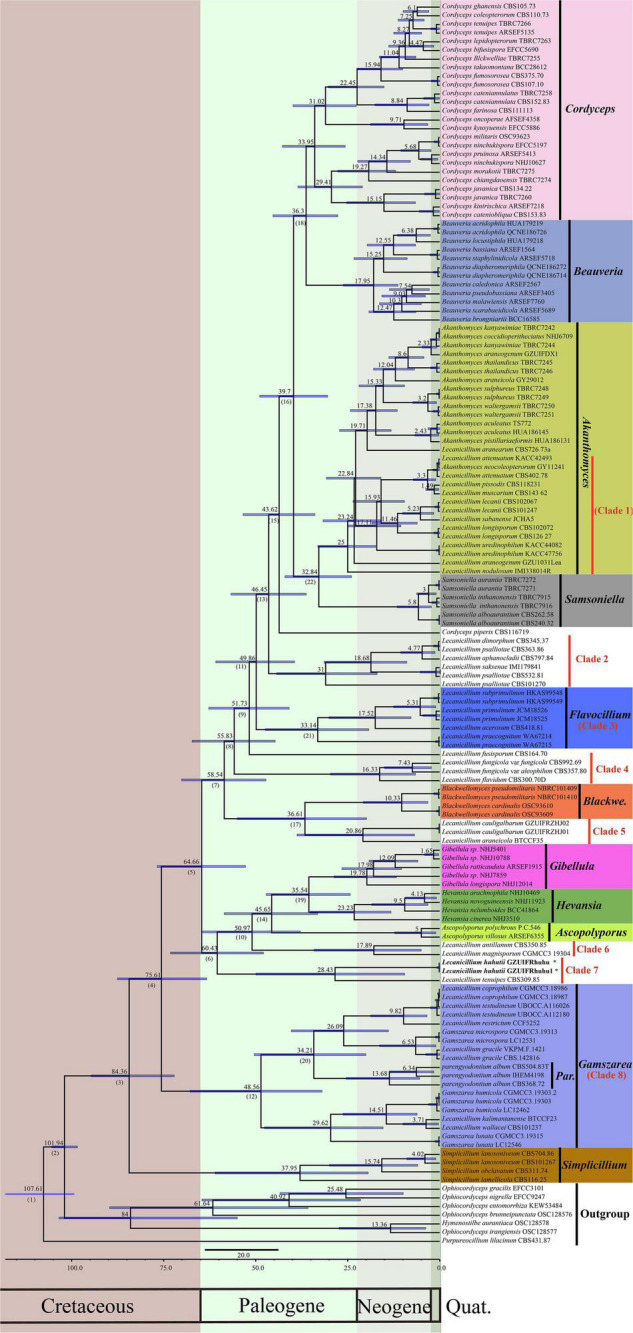
Maximum clade credibility chronogram of *Lecanicillium* in Cordycipitaceae evolution. The chronogram results from the BEAST analysis are based on the topology of the ML tree. Each node represents the mean divergence time estimate (displayed above the nodes), while bars show the 95% HPD. Numbers corresponding to dated groups shown in Table 1 are indicated in square brackets above the nodes. The proposed novel species of the genus *Lecanicillium* is indicated in bold font and marked with “*”.

To explore the evolution of internal phylogeny, origin, and evolutionary history of *Lecanicillium*, all species of *Lecanicillium* and other members of the Cordycipitaceae based on previous phylogenetic studies were selected. The clustering of taxa of genera in the family Cordycipitaceae (Figure 2) almost matched the topology of the resulting consensus phylogenetic tree (Figure 1). Dating analyses supported the supposition that the ancestor of the genus *Lecanicillium* was in the Cretaceous period (84.36 Mya, 95% HPD: 72.12–94.74 Mya). After originating from a common ancestor, eight clades of *Lecanicillium* were derived and evolved independently in parallel with other genera of Cordycipitaceae (Figure 2 and Table 1). Based on the clear divergence age estimates, *Lecanicillium* clade 8 originated earlier as an independent group in the Cretaceous period (75.61 Mya, 95% HPD: 63.31–87.54 Mya), while *Lecanicillium* clades 1–7 originated later as an independent group in the boundary of the Cretaceous and Paleogene periods (64.66 Mya, 95% HPD: 52.75–76.74 Mya). These analyses also provided a clear direction for the attribution of *Lecanicillium* (Figure 2 and Table 1).

**TABLE 1:** Mean and range (95% HPD) divergence time estimations (Mya) of the genus *Lecanicillium* in the family Cordycipitaceae.

Node	Mean (Mya)	95% HPD (Mya)	Genus
1	107.61	99.29–125.33	(2)
2	101.94	98.43–105.42	(3)
3	84.36	72.12–94.74	(4)/*Simplicillium*
4	75.61	63.31–87.54	(5)/***Lecanicillium* clade 8** (*Gamszarea*)
5	64.66	52.75–76.74	(6)/(7)
6	60.43	47.88–73.14	(10)/***Lecanicillium* clade 7**
7	58.54	47.21–70.13	(8)/(17)
8	55.83	44.71–67.28	(9)/***Lecanicillium* clade 4**
9	51.73	40.90–62.74	(11)
10	50.97	37.85–64.63	(14)/***Lecanicillium* clade 6**
11	49.86	39.46–60.85	(13)/***Lecanicillium* clade 3** (*Flavocillium*)
12	48.56	31.95–67.86	(20)
13	46.45	36.25–56.71	(15)/***Lecanicillium* clade 2**
14	45.64	33.15–58.54	*Ascopolyporus*/(19)
15	43.62	33.90–53.40	(16)
16	39.70	30.44–48.91	(18)/(22)
17	36.61	19.89–55.02	*Blackwellomyces/* ***Lecanicillium* clade 5**
18	36.30	27.68–45.30	*Cordyceps*/*Beauveria*
19	35.54	24.30–47.13	*Hevansia*/*Gibellula*
20	34.21	20.07–50.39	*Parengyodontium*
21	33.14	19.27–47.38	–
22	32.84	23.95–42.05	*Samsoniella*/*Akanthomyces*/ ***Lecanicillium* clade 1**

*The bolded terms mean the summarized Lecanicillium clade.*

### Taxonomy

***Lecanicillium huhutii* X. Zou and Y.M. Zhou, sp.nov-MycoBank: MB835961**

Etymology: The epithet “*huhutii*” refers to the infant name of the discoverer (a 4-year-old boy).

Type: China. Guizhou Province: Guiyang City, Huaxi park, 5 Oct 2013, Sequences from isolated strain GZUIFRhuhu have been deposited in GenBank (ITS: MN944445; *TEF*: MT006068; *RPB1*: MT006058; *RPB2*: MT006063). The information of each sequence of strain GZUIFRhuhu1 was the same as that of strain GZUIFRhuhu.

**FIGURE 3:**
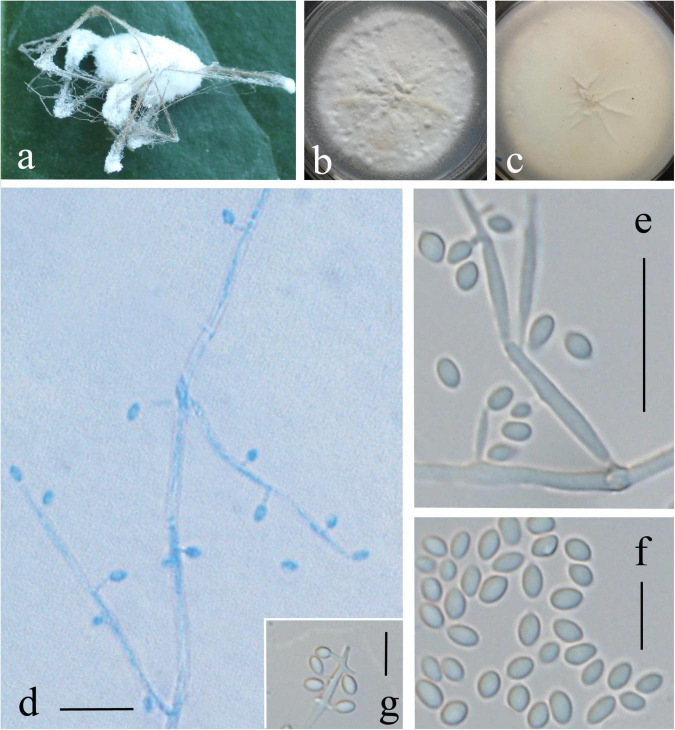
*Lecanicillium huhutii*. **(a)** Synnemata emerged from the corpse of a spider. **(b,c)** Culture plate, cultured on PDA medium. **(d–g)** Conidiophores, conidiogenous cells, and conidia. Bars: **(d,e)** = 10 mm; **(f,g)** = 5 μm.

Description: Colonies on PDA are 72 mm in diameter after 20 days at 25°C, and circular, white, partly yellowish, and reverse yellowish-white. Mycelia are 0.9–1.8 μm, wide, hyaline, and smooth. There are two kinds of conidiogenous cells: (1) arising from aerial hyphae, solitary or 2–3 whorls, 3.5–12 × 0.5–1 μm; (2) very short, arising from conidiogenous cells or aerial hyphae, 0.7–1.2 × 0.3–0.5 μm. Conidia are oval, aseptate, and 1.5–2.4 × 1–1.5 μm. In culture, both phialides and conidia are of similar general shape and size to those found on the host stemborer (Figure 3).

Host: Spider

Habitat and distribution: Hidden in a hole of a tree in Huaxi park of Guizhou province, China.

Teleomorph: Not known.

Remarks: Regarding phylogenetic relationships, *L. huhutii* is closely related to *L. tenuipes*. The two strains (GZUIFRhuhu and GZUIFRhuhu1) formed a distinct lineage. All species of *Lecanicillium* were included in the phylogenetic analysis except for *L. evansii* for which sequence data could not be located in public databases, although Zare and Gams (2001) published ITS sequences. The morphological features of *L. evansii* include brownish-cream to brown reverse colonies and two types of conidia, slightly falcate with pointed end macroconidia, 4.5–7.5 × 0.8–1.2 μm, and slightly curved microconidia, 2.0–3.0 × 0.8–1.2 μm (Zare and Gams, 2001). *L. evansii* is distinct from *L. huhutii*, which has one type of conidia (1.5–2.4 × 1–1.5 μm) and two types of phialides.

Regarding the morphology of *Lecanicillium*, only *L. dimorphum* is reported to have two kinds of conidiogenous cells. However, *L. huhutii* can be clearly distinguished by the size of the phialides (A, 14–30 × 1.0–1.5 μm; B, 5–12 × 0.7–1.5 μm) and conidia (A, 6–11 × 1.5–2.5 μm; B, 2.5–4.5 × 1.0–1.5 μm) (Zare and Gams, 2001).

## Discussion

The genus *Lecanicillium* was previously shown to be a polyphyletic group (Zare and Gams, 2001; Kepler et al., 2017), which is supported by the phylogeny in the current study. Eight clades of *Lecanicillium* were derived and evolved independently in parallel with other genera of the family Cordycipitaceae. The *Lecanicillium* species were transferred to *Akanthomyces* by Kepler et al. (2017) and were all included in *Lecanicillium* clade 1 based on the phylogeny in the current study. All other species in this group (*Lecanicillium pissodis*, *L. araneogemum*, *L. uredinophilum*, and *L. nodulosum*) should be transferred to *Akanthomyces*, as mentioned in our previous work (Zhou et al., 2018). Significantly, all *Lecanicillium* clade 1 members are distinct from other members of the genus *Akanthomyces*, with the *Lecanicillium* clade 1 members possess a relatively uniform sporulation structure of globose heads with a higher number of conidia.

As more members of the genus are discovered in the future, it will be interesting to ascertain whether *Lecanicillium* clade 1 should be re-established as a separate genus. Alternatively, it may be worth revisiting this clade in detail, as in the case of *Isaria*, which was rejected by the same recommendation of (Kepler et al., 2017; Mongkolsamrit et al., 2018). Zhang et al. (2020) transferred five species of *Lecanicillium* to a novel genus with the name *Gamszarea*, which is also supported by the phylogeny of *Lecanicillium* clade 8 in the current study. Significantly, the genus *Parengyodontium* is included in the *Gamszarea*. Considering *Parengyodontium album* is an important human pathogen (Tsang et al., 2016), it will be interesting to determine whether other species of the genus *Gamszarea* can also cause human disease, warranting further investigation in the future. In addition, the phylogeny in the current study indicates that *L. gracile* (Ponizovskaya et al., 2020) should be transferred to the genus *Gamszarea*. Wang et al. (2020) transferred three species of *Lecanicillium* to a new genus as *Flavocillium* based on ITS sequence data. This is also supported by the phylogeny in the current study, with *Lecanicillium* clade 3 including *L. pracecognitum* (Crous et al., 2020). Significantly, the differences in the divergence time estimations of *Lecanicillium* clades 2-5 were smaller compared with those of other genera of the Cordycipitaceae. Considering the morphological and structural similarities of *Lecanicillium* clade 2-5, members of these clades are likely to change as more new species are discovered in the future. Therefore, more species are needed to resolve the attribution problem of *Lecanicillium* spp.

In the current study, a direct relationship between *Lecanicillium* and the Cretaceous-Tertiary extinction event was not detected (Schulte et al., 2010). This suggests that the diversity of *Lecanicillium* is more likely to be caused by long-term environmental adaptation and coevolution with insects, rather than by dramatic extinction events. In the Cretaceous period, the radiation of diverse monocot and eudicot lineages began to change the organisms that interacted with each other, including diversification of the pollinating insects, phytophagous insects, and entomogenous fungi (Labandeira and Sepkoski, 1993; Soltis and Soltis, 2004; Sung et al., 2008).

Spider-pathogenic fungi are widely distributed globally. The first records of spider-pathogenic fungi appear in 1856 (Gray, 1858). Evans and Samson (1987) note that all confirmed and unconfirmed parasitic spider fungi belong to the order Hypocreales. Interestingly, spider-pathogenic fungi are almost restricted to the families of Cordycipitaceae and Ophiocordycipitaceae within this order, with no reports of any spider-pathogenic fungi belonging to Clavicipitaceae within Hypocreales (Shrestha et al., 2019). Statistically, spider-pathogenic fungi are predominantly concentrated in the family Cordycipitaceae of Hypocreales with 8 genera and 75 species out of 13 genera and 86 species (Shrestha et al., 2019). Among them, there are 5 spider-pathogenic species of *Lecanicillium*, including *L. lecanii*, *L. tenuipes*, *L. aranearum* (Zare and Gams, 2001), *L. araneicola* (Sukarno et al., 2009), and *L. araneogenum* (Chen et al., 2017). With the readjustment of the Cordycipitaceae, spider pathogens with *Cordyceps*-, *Isaria*-, *Lecanicillium*-, or *Torrubiella*-like morphs have been described in the genus *Akanthomyces* (Kepler et al., 2017). However, since the taxonomic status of the genus *Lecanicillium* has not been completely solved, only *L. lecanii* and *L. araneogenum* have been placed into *Akanthomyces* at present. In addition, Kepler’s suggestion to re-use the name *Engyodontium* is another problem to consider. According to previous descriptions, *L. aranearum* (Petch) Zare and W. Gams (≡ *Cephalosporium aranearum* Petch, = *Engyodontium arachnophilum*), associated with *Torrubiella alba* Petch, and *L*. *tenuipes* (Petch) Zare and W. Gams (≡ *Acremonium tenuipes* Petch, = *Engyodontium aranearum*) (Shrestha et al., 2019). Given that there is only one recognized species of *Engyodontium*, which is *E. parvisporum* (Kepler et al., 2017), whether these two kinds of appellation (*L. tenuipes* and *L. aranearum*) should be restired requires further discussion. Although the novel species described in the current study is close to *L. tenuipes* in the phylogenetic tree, we think this species is clearly distinguished from *Engyodontium* by morphology. We also treat the novel species as *Lecanicillium*, considering the small sample and the unknown teleomorph. Thus, based on the present molecular phylogeny, derived from nuclear and ribosomal DNA sequence data, together with morphological evidence, a distinct novel species of the genus *Lecanicillium* is proposed, *L. huhutii*, a new spider-pathogenic fungus.

## Conclusion

The pivotal internal phylogeny, origin, and evolutionary history of *Lecanicillium* in the family Cordycipitaceae were studied. Phylogenetic and morphological analyses indicated there were eight representative clades (four representative branches of evolutionary history), including clade 1 (members have a relatively uniform sporulation structure comprising globose heads with a higher number of conidia), clade 8 (including all members of *Gamszarea*), clades 2–5 (the differences in divergence time estimations were smaller for these clades compared with other clades), and clades 6–7 (members are close to *Gibellula*, *Hevansia* and *Ascopolyporus*). *L. pissodis*, *L. araneogemum*, *L. uredinophilum*, and *L. nodulosum* in clade 1 should be transferred to the genus *Akanthomyces*. The monotypic genus *Parengyodontium* should be merged with the genus *Gamszarea*. More new species need to be discovered to thoroughly resolve the attribution problem of *Lecanicillium*. Finally, no major lineages of *Lecanicillium* were correlated with the nearby Cretaceous-Tertiary extinction event, indicating that the diversity of *Lecanicillium* is more likely to be caused by long-term environmental adaptation and coevolution with insects rather than by dramatic extinction events.

## Data Availability Statement

The datasets presented in this study can be found in online repositories. The names of the repository/repositories and accession number(s) can be found below: NCBI GenBank, accession numbers: ITS: MN944445; TEF: MT006068; RPB1: MT006058; RPB2: MT006063.

## Author Contributions

Y-MZ performed the experiments, analyzed the data, and wrote the article. J-RZ revised the manuscript. J-JQ analyzed the data. XZ isolated the fungus and revised the manuscript. All authors reviewed the manuscript.

## Conflict of Interest

The authors declare that the research was conducted in the absence of any commercial or financial relationships that could be construed as a potential conflict of interest.

## Publisher’s Note

All claims expressed in this article are solely those of the authors and do not necessarily represent those of their affiliated organizations, or those of the publisher, the editors and the reviewers. Any product that may be evaluated in this article, or claim that may be made by its manufacturer, is not guaranteed or endorsed by the publisher.

## Footnotes

1
http://www.indexfungorum.org

2
https://www.ncbi.nlm.nih.gov/genbank/

## Abbreviations

GZAC: Institute of Fungal Resources of Guizhou University
ITS: ITS1-5.8S rDNA-ITS2 region the first and the internal transcribed spacers
LSU: Large Subunits of the rDNA
NCBI: National Center for Biotechnology Information
PDA: potato dextrose agar
RPB1: DNA-directed RNA polymerase II subunit rpb1
RPB2: DNA-directed RNA polymerase II subunit rpb2
SSU: small subunits of the rDNA
TEF: transcription elongation factor-1 α .
