# Supplementary material for: Estimated Divergence Times of Lecanicillium in the Family Cordycipitaceae Provide Insights Into the Attribution of Lecanicillium
Source: Front Microbiol. 2022 May 6;13:859886. doi: 10.3389/fmicb.2022.859886 (PMC9121009; doi:10.3389/fmicb.2022.859886)
Supplement: Supplementary file 1 [file Table_1.DOCX]

| **Additional** **Table1.** Specimen information and GenBank accession numbers used in this study | | | | | |
| --- | --- | --- | --- | --- | --- |
| Species | Voucher Information | ITS | *TEF* | *RPB1* | *RPB2* |
| *Akanthomyces aculeatus* | HUA 186145 |  | MF416465 |  |  |
| *A. aculeatus* | TS 772 | KC519371 | KC519366 |  |  |
| *A. araneicola* | GY 29012 | MK942434 | MK955950 | MK955945 | MK955948 |
| *A. araneogenum* | GZUIF DX1 | KU893152 |  | MH978181 | MH978184 |
| *A. coccidioperitheciatus* | NHJ 6709 | JN049865 | EU369025 | EU369067 | EU369086 |
| *A. kanyawimiae* | TBRC 7242 | MF140751 | MF140838 | MF140784 | MF140808 |
| *A. kanyawimiae* | TBRC 7244 | MF140752 | MF140836 |  |  |
| *A. neocoleopterorum* | GY11241 | MN093295 | MN097813 | MN097816 | MN097812 |
| *A. pistillariaeformis* | HUA 186131 |  | MF416466 |  |  |
| *A. sulphureus* | TBRC 7248 | MF140758 | MF140843 | MF140787 | MF140812 |
| *A. sulphureus* | TBRC 7249 | MF140757 | MF140842 | MF140786 | MF140734 |
| *A. thailandicus* | TBRC 7246 | MF140755 | MF140840 |  | MF140810 |
| *A. thailandicus* | TBRC 7245 | MF140754 | MF140839 |  | MF140809 |
| *A. waltergamsii* | TBRC 7250 | MF140749 | MF140835 |  |  |
| *A. waltergamsii* | TBRC 7251 | MF140747 | MF140833 | MF140781 | MF140805 |
| *Ascopolyporus villosus* | ARSEF 6355 |  | DQ118750 | DQ127241 |  |
| *Asc. polychrous* | P.C. 546 |  | DQ118745 | DQ127236 |  |
| *Beauveria acridophila* | QCNE 186726 | JQ958605 | JQ958618 | JX003855 | JX003844 |
| *Bea.* *acridophila* | HUA 179219 |  | JQ958613 | JX003857 | JX003841 |
| *Bea. bassiana* | ARSEF 1564 | HQ880761 | HQ880974 | HQ880833 | HQ880905 |
| *Bea. brongniartii* | BCC 16585 | JN049867 | JF416009 | JN049885 | JF415991 |
| *Bea. caledonica* | ARSEF 2567 | HQ880817 | EF469057 | EF469086 | HQ880961 |
| *Bea. diapheromeriphila* | QCNE 186714 | JQ958603 | JQ958611 | JX003850 |  |
| *Bea. diapheromeriphila* | QCNE 186272 | JQ958599 | JQ958610 | JX003848 |  |
| *Bea. locustiphila* | HUA 179218 | JQ958606 | JQ958619 | JX003846 | JX003845 |
| *Bea. malawiensis* | ARSEF 7760 |  | DQ376246 | HQ880897 | HQ880969 |
| *Bea. pseudobassiana* | ARSEF 3405 | AY532022 | AY531931 | HQ880864 | HQ880936 |
| *Bea. scarabaeidicola* | ARSEF 5689 | JN049827 | DQ522335 | DQ522380 | DQ522431 |
| *Bea. staphylinidicola* | ARSEF 5718 |  | EF468776 | EF468881 |  |
| *Blackwellomyces cardinalis* | OSC 93610 | JN049843 | EF469059 | EF469088 | EF469106 |
| *Bla. cardinalis* | OSC 93609 |  | DQ522325 | DQ522370 | DQ522422 |
| *Bla. pseudomilitaris* | NBRC 101409 | JN943305 |  | JN992482 |  |
| *Bla. pseudomilitaris* | NBRC 101410 | JN943307 |  | JN992481 |  |
| *Cordyceps bifusispora* | EFCC 5690 |  | EF468746 | EF468854 | EF468909 |
| *C. cateniannulata* | CBS 152.83 | AY624172 | JQ425687 |  |  |
| *C. cateniobliqua* | CBS 153.83 | AY624173 | JQ425688 |  | MG665236 |
| *C. chiangdaoensis* | TBRC 7274 | KT261393 | KT261403 |  |  |
| *C. coleopterorum* | CBS 110.73 | AY624177 | JF416028 | JN049903 | JF416006 |
| *C. farinosa* | CBS 111113 | AY624181 | GQ250022 |  | GU979973 |
| *C. fumosorosea* | CBS 107.10 | AY624184 | HM161735 |  | MG665237 |
| *C. fumosorosea* | CBS 375.70 | AY624183 | HM161736 |  | MG665238 |
| *C. ghanensis* | CBS 105.73 | AY624185 |  |  |  |
| *C. javanica* | CBS 134.22 | AY624186 | JQ425683 |  |  |
| *C. kintrischica* | ARSEF 7218 | EU553278 | GU734751 |  |  |
| *C. kyusyuensis* | EFCC 5886 |  | EF468754 | EF468863 | EF468917 |
| *C. militaris* | OSC 93623 |  | EF468762 | EF468869 |  |
| *C. morakotii* | TBRC 7275 | KT261388 | KT261398 |  |  |
| *C. ninchukispora* | EFCC 5197 |  | EF468760 | EF468868 |  |
| *C. ninchukispora* | NHJ 10627 |  | EF468763 | EF468870 |  |
| *C. oncoperae* | AFSEF 4358 |  | EF468785 | EF468891 | EF468936 |
| *C. pruinosa* | ARSEF 5413 | JN049826 | DQ522351 | DQ522397 | DQ522451 |
| *C. takaomontana* | BCC 28612 | FJ765285 | FJ765268 |  |  |
| *C. tenuipes* | ARSEF 5135 | AY624196 | JF416020 | JN049896 | JF416000 |
| *C. tenuipes* | TBRC 7266 | MF140742 | MF140828 | MF140777 | MF140801 |
| *C. blackwelliae* | TBRC 7255 | MF140737 | MF140823 | MF140772 | MF140796 |
| *C. cateniannulatus* | TBRC 7258 | MF140753 | MF140850 | MF140767 |  |
| *C. javanica* | TBRC 7260 | MF140744 | MF140830 | MF140779 | MF140803 |
| *C. lepidopterorum* | TBRC 7263 | MF140765 | MF140819 | MF140768 | MF140792 |
| *Gamszarea humicola* | CGMCC3.19303 | MK329092 | MK336027 |  | MK335979 |
| *Gam. humicola* | LC12462 | MK329093 | MK336028 |  | MK335980 |
| *Gam. lunata* | CGMCC3.19315 | MK329094 | MK336029 |  | MK335981 |
| *Gam. lunata* | LC12546 | MK329095 | MK336030 |  | MK335982 |
| *Gam. microspora* | CGMCC3.19313 | MK329096 | MK336031 |  | MK335983 |
| *Gam. microspora* | LC12531 | MK329097 | MK336032 |  | MK335984 |
| *Gibellula longispora* | NHJ 12014 |  | EU369017 | EU369055 | EU369075 |
| *G.* sp. | NHJ 7859 |  |  | EU369064 | EU369085 |
| *G.* sp. | NHJ 10788 |  | EU369019 | EU369058 | EU369078 |
| *G.* sp. | NHJ 5401 |  |  | EU369059 | EU369079 |
| *G. ratticaudata* | ARSEF 1915 | JN049837 | DQ522360 | DQ522408 | DQ522467 |
| *Hevansia nelumboides* | BCC 41864 | JN201871 | JN201867 |  |  |
| *Hev. novoguineensis* | NHJ 11923 |  | EU369013 | EU369052 | EU369072 |
| *Hev. arachnophila* | NHJ 10469 |  | EU369008 | EU369047 |  |
| *Hev. cinerea* | NHJ 3510 |  | EU369009 | EU369048 | EU369070 |
| *Lecanicillium acerosum* | CBS418.81 | EF641893 | KM283810 | KM283832 | KM283852 |
| *L. antillanum* | CBS350.85 | AJ292392 | DQ522350 | DQ522396 | DQ522450 |
| *L. aphanocladii* | CBS797.84 |  | KM283811 | KM283833 | KM283853 |
| *L. aranearum* | CBS726.73a | AJ292464 | EF468781 | EF468887 | EF468934 |
| *L. araneicola* | BTCC-F35 | AB378506 |  |  |  |
| *L. araneogenum* | GZU1031Lea |  | KX845697 | KX845699 | KX845701 |
| *L. attenuatum* | CBS402.78 | AJ292434 | EF468782 | EF468888 | EF468935 |
| *L. attenuatum* | KACC42493 |  | KM283804 | KM283826 | KM283846 |
| *L.* *cauligalbarum* | GZUIFRZHJ01 | MH730663 | MH801920 | MH801922 | MH801924 |
| *L. cauligalbarum* | GZUIFRZHJ02 | MH730664 | MH801921 | MH801923 | MH801925 |
| *L. coprophilum* | CGMCC3.18986 | MH177616 | MH184587 | MH177622 | MH177624 |
| *L. coprophilum* | CGMCC3.18987 | MH177615 | MH184586 | MH177621 | MH177623 |
| *L. dimorphum* | CBS345.37 |  | KM283812 | KM283834 | KM283854 |
| *L. flavidum* | CBS300.70D | EF641877 | KM283813 |  | KM283855 |
| *L. fungicola var. aleophilum* | CBS357.80 | NR_111064 | KM283815 | KM283835 | KM283856 |
| *L. fungicola var. fungicola* | CBS992.69 | NR_119653 | KM283816 |  | KM283857 |
| *L. fusisporum* | CBS164.70 | AJ292428 | KM283817 | KM283836 | KM283858 |
| *L. gracile* | CBS 142816 | LT598647 |  |  |  |
| *L. gracile* | VKPM F-1421 | MF682449 |  |  |  |
| ***L. huhutii*** | **GZUIFRhuhu** | **MN944445** | **MT006068** | **MT006058** | **MT006063** |
| *L. kalimantanense* | BTCC-F23 | AB360356 |  |  |  |
| *L. lecanii* | CBS101247 | JN049836 | DQ522359 | KM283837 | KM283859 |
| *L. lecanii* | CBS102067 |  | KM283818 | KM283838 | KM283860 |
| *L. longisporum* | CBS102072 |  | KM283819 | KM283839 | KM283861 |
| *L. longisporum* | CBS126.27 |  | KM283820 | KM283840 | KM283862 |
| *L. magnisporum* | CGMCC3.19304 | MK329102 | MK336037 |  | MK335985 |
| *L. muscarium* | CBS143.62 |  | KM283821 | KM283841 | KM283863 |
| *L. nodulosum* | IMI 338014R | EF513012 |  |  |  |
| *L. pissodis* | CBS118231 |  | KM283822 | KM283842 | KM283864 |
| *L. praecognitum* | WA67214 | MT247059 | MT267524 |  | MT267526 |
| *L. praecognitum* | WA67215 | MT247058 | MT267523 |  | MT267525 |
| *L. primulinum* | JCM 18525 | AB712266 |  |  |  |
| *L. primulinum* | JCM 18526 | AB712267 |  |  |  |
| *L. psalliotae* | CBS532.81 | JN049846 | EF469067 | EF469096 | EF469112 |
| *L. psalliotae* | CBS101270 |  | EF469066 | EF469095 | EF469113 |
| *L. psalliotae* | CBS363.86 |  | EF468784 | EF468890 |  |
| *L. restrictum* | CCF5252 | LT548279 | LT626943 |  |  |
| *L. sabanense* | JCHA5 | KC633232 | KC633266 |  | KC633249 |
| *L. saksenae* | IMI 179841 | AJ292432 |  |  |  |
| *L. subprimulinum* | HKAS99548 | MG585314 | MG585317 |  |  |
| *L. subprimulinum* | HKAS99549 | MG585318 | MG585321 |  |  |
| *L. testudineum* | UBOCC-A112180 | LT992874 | LT992868 |  |  |
| *L. testudineum* | UBOCC-A116026 | LT992871 | LT992867 |  |  |
| *L. tenuipes* | CBS309.85 | JN036556 | DQ522341 | KM283844 | KM283866 |
| *L. uredinophilum* | KACC44082 |  | KM283806 | KM283828 | KM283848 |
| *L. uredinophilum* | KACC47756 |  | KM283807 | KM283829 | KM283849 |
| *L. wallacei* | CBS101237 | EF641891 | EF469073 | EF469102 | EF469119 |
| *Parengyodontium album* | CBS368.72 | LC092891 |  |  |  |
| *Par. album* | CBS504.83T | LC092880 |  |  |  |
| *Par. album* | IHEM4198 | LC092887 | DQ268655 |  |  |
| *Purpureocillium lilacinum* | CBS 431.87 | AY624188 | EF468791 | EF468897 | EF468940 |
| *Samsoniella inthanonensis* | TBRC 7915 | MF140761 | MF140849 | MF140790 | MF140815 |
| *Sam. inthanonensis* | TBRC 7916 | MF140760 | MF140848 | MF140789 | MF140814 |
| *Sam. aurantia* | TBRC 7271 | MF140764 | MF140846 | MF140791 | MF140818 |
| *Sam. aurantia* | TBRC 7272 | MF140763 | MF140845 |  | MF140817 |
| *Sam. alboaurantium* | CBS 240.32 | AY624178 | JF416019 | JN049895 | JF415999 |
| *Sam. alboaurantium* | CBS 262.58 | MH857775 | JQ425685 | MF416654 | MF416448 |
| *Simplicillium lamellicola* | CBS 116.25 | AJ292393 | DQ522356 | DQ522404 | DQ522462 |
| *Sim. lanosoniveum* | CBS 704.86 | AJ292396 | DQ522358 | DQ522406 | DQ522464 |
| *Sim. lanosoniveum* | CBS 101267 | AJ292395 | DQ522357 | DQ522405 | DQ522463 |
| *Sim. obclavatum* | CBS 311.74 |  | EF468798 |  |  |
